# Cavernous hemangioma in the masticatory space

**DOI:** 10.31744/einstein_journal/2024RC1106

**Published:** 2024-11-19

**Authors:** Marina Nahas Dafico Bernardes, Lucas da Silva Braz, Júlia Ramos de Melo, Cárita Lopes Macêdo, Jordanna Ferreira Lousek, Hugo Fernandes de Paula, Hugo Valter Lisboa Ramos, Claudiney Candido Costa

**Affiliations:** 1 Centro Estadual de Reabilitação e Readaptação Dr. Henrique Santillo Goiânia GO Brazil Centro Estadual de Reabilitação e Readaptação Dr. Henrique Santillo, Goiânia, GO, Brazil.

**Keywords:** Hemangioma, cavernous, Hemangioma, Nose deformities, acquired, Arteriovenous malformations, Vascular neoplasms, Turbinates

## Abstract

Hemangiomas are benign congenital vascular tumors that commonly arise in the head and neck regions. Although they present with indolent growth and involution in most cases, they can cause facial deformities. Hemangiomas have three subtypes: capillary, cavernous, and mixed. The cavernous type is most commonly observed on the lateral wall of the nose or the inferior turbinate. This report describes a case of cavernous hemangioma diagnosed in a rare location in the left retromaxillary masticatory space in a 67-year-old woman. Total excision of the lesion was performed with surgical treatment through the Caldwell-Luc access. Anatomopathological and immunohistochemical examinations suggested a malformative vascular lesion with a cavernous hemangioma pattern and without signs of malignancy.

## INTRODUCTION

Hemangiomas are congenital benign tumors that originate from the vascular tissue of the skin, mucosa, muscle, salivary gland, and bones and are histologically classified as bone and non-osseous. Although lesions are common in the head and neck regions, the majority arise in the vestibule or nasal septum and are rarely observed in the paranasal sinuses or their borders, with the latter being more frequently cavernous.^(1)^ These indolent lesions grow slowly and may cause deformities in the patient.^(2)^ In most cases, they are diagnosed at birth and commonly persist into adulthood. There are three main subtypes of these lesions: capillary, cavernous, and mixed.^(3)^

Information regarding this tumor is scarce in the literature, and its etiology remains unclear.^(4)^ This study reports a rare case of left retromaxillary masticatory space involvement in a cavernous hemangioma in a 67-year-old woman.

## CASE REPORT

**Figure 1:**
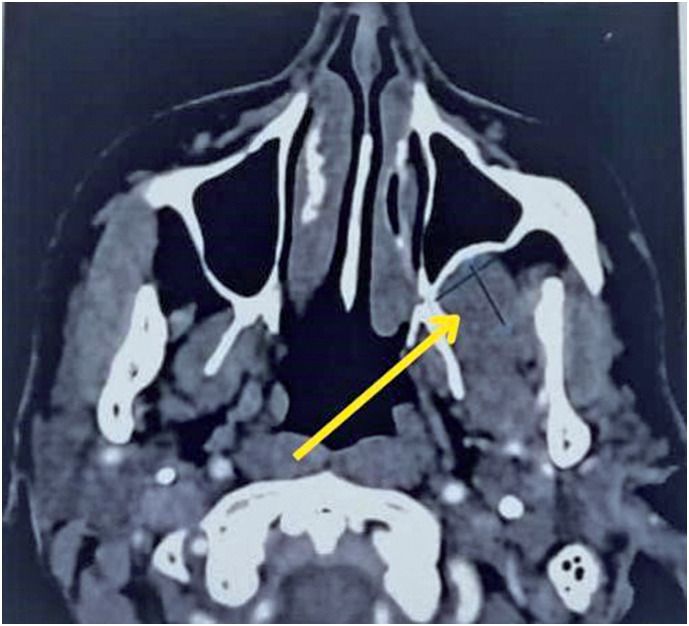
Computed tomography image of the sinuses in a bone window in an axial section, with the yellow arrow indicating a well-defined nodular lesion measuring approximately 2.0cm in diameter

**Figure 2:**
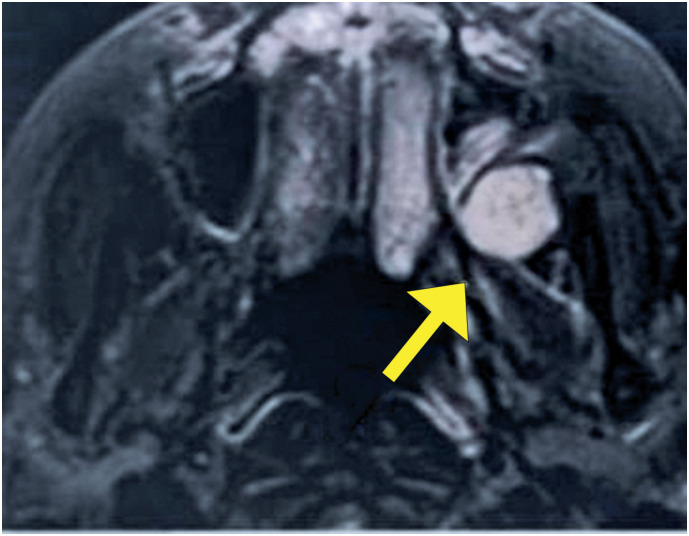
Skull magnetic resonance imaging in an axial section reveals a nodular, solid lesion in the masticatory space on the left, posterior to the ipsilateral maxillary sinus, with a volume of 2.0 × 1.8cm and hyperintensity on T2/ fluid-attenuated inversion recovery

**Figure 3:**
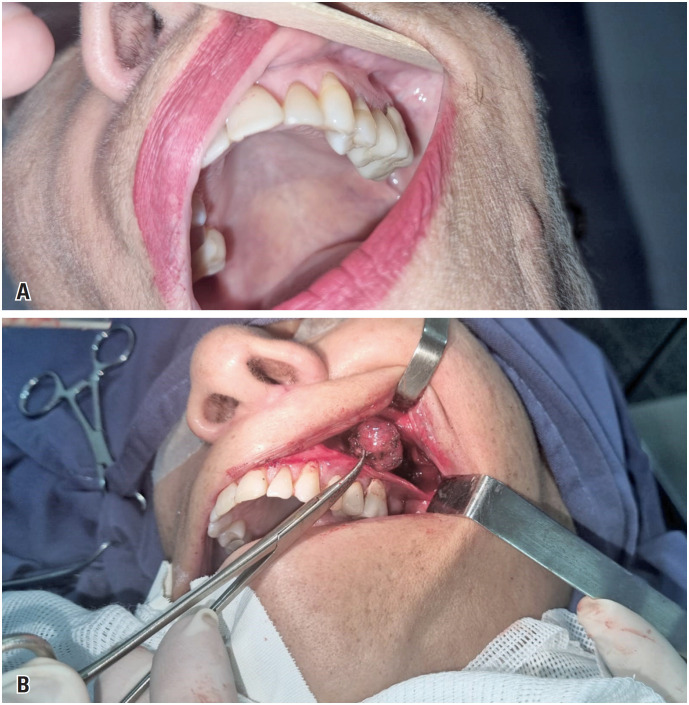
(A) View of the gingivolabial groove region on the left where the incision will be made via Caldwell-Luc to remove the tumor; (B) Intraoperative photo showing tumor excision

The patient was a 67-year-old woman with a history of breast cancer treated with surgical resection, radiotherapy, and chemotherapy in 2021, without recurrence. She denied previous surgical otorhinolaryngological treatments or a history of head and neck trauma. She was admitted to the Otorhinolaryngology and Head and Neck Surgery clinic, without symptomatic complaints but with the following finding on routine imaging examinations: a lesion in the left retromaxillary region with bulging of the posterior wall of the ipsilateral maxillary sinus on computed tomography (CT) (Figure 1) and a nodular, solid lesion in the chewing space on the left, posterior to the ipsilateral maxillary sinus, with a volume of 2.0 × 1.8cm and hyperintensity on T2/fluid-attenuated inversion recovery skull magnetic resonance imaging (MRI) (Figure 2). Physical examination revealed no bulging of the face or cheek region on the left side. There was no pain on palpation and no history of secretory drainage or fistulization. The lesion underwent total excision through the Caldwell-Luc access, with an incision in the left buccal mucosa and access to the left retromaxillary space; exposure and total removal of the lesion were achieved without complications and preoperative embolization (Figures 3A and 3B). The specimen was sent for anatomopathological examination, which revealed a cavernous hemangioma without signs of malignancy. The patient remained under regular follow-up without recurrence and was asymptomatic.

The study was approved by the *Associação Goiana de Integralização e Reabilitação,* CAAE: 19960619.8.0000.5082; #6494309.

## DISCUSSION

Cavernous hemangiomas are rare lesions generally found in the bone septum or lateral nasal wall and affect two women for every man, generally in their fourth decade of life. Its etiology remains unknown; however, a history of local trauma is considered a possible cause.^(5,6)^ The clinical presentation varies depending on the volume and location of the lesion. The cavernous subtype is characterized by lesions that are poorly defined in terms of depth and are generally subcutaneous. Patients’ main complaint is the growth of a mass in the head and neck regions, often painless and presenting with headaches. Epistaxis and nasal obstruction are rare.^(2)^ Imaging examinations are necessary for diagnosis and reveal findings common to benign lesions. Computed tomography of the facial sinuses demonstrates well-defined lesions with soft tissue density, which impose remodeling on the cortical bone and enhancement after contrast. MRI shows isointense or hypointense lesions on T1 and hyperintense lesions on T2, with post-contrast intensification. Other options for diagnosis and treatment include angiography and transarterial embolization.^(3)^

Differential diagnoses include epidermoid cysts, meningoceles, carcinomas, melanomas, and other bone metastases, such as osteosarcoma and fibrous dysplasia. Other tumors included angiofibroma chordomas, hemangiovenous hemangioendothelioma, angiomatous glomus tumors, lymphangioma, hemangioperistioma, and hemangiosarcoma.^(2-4)^ The recommended treatment is surgical excision, usually through Caldwell-Luc, with an incision in the gingivolabial groove, extensive removal of the tumor and the underlying mucosa, and cauterization or ligation of the feeding vessels. Preoperative embolization is necessary in cases of extensive and highly vascularized lesions. Complications of this technique include recurrent sinusitis, pain or numbness in the face and teeth, facial swelling, and damage to the infraorbital nerves and vessels.^(3,6-8)^

## CONCLUSION

Cavernous hemangiomas are benign vascular tumors that should be considered in diagnostic investigations of head and neck tumor lesions. This report presents a rare case of cavernous hemangioma located in the masticatory space, with few reports in the literature contributing to the dissemination of scientific knowledge about this tumor.

Imaging examinations strongly contribute to the development of diagnostic hypotheses and surgical planning; however, only anatomopathological examinations provide a definitive diagnosis. The recommended treatment is surgery, preferably conservative.
